# Sampling for DUS Test of Flower Colors of *Ranunculus asiaticus* L. in View of Spatial and Temporal Changes of Flower Colorations, Anthocyanin Contents, and Gene Expression Levels

**DOI:** 10.3390/molecules24030615

**Published:** 2019-02-10

**Authors:** Yanfang Liu, Tao Lin, Lijuan Du, Jiangmin Wang, Xiaohong Yang, Jianhua Zhang, Peng Zhang, Yangang Li, Junfeng Shi, Xuhong Yang

**Affiliations:** 1 Quality Standard and Testing Technology Research Institute, Yunnan Academy of Agricultural Sciences, Kunming 650205, China; liuyf528@163.com (Y.L.); lintaoj@126.com (T.L.); 15825298061@163.com (L.D.); jiangm_wang@163.com (J.W.); yangxhjq@163.com (X.Y.); eapvpf2@163.com (J.Z.); peterzhng@126.com (P.Z.); 2 Development Center of Science and Technology, Ministry of Agriculture, Beijing 100122, China

**Keywords:** *Ranunculus asiaticus* L., color phenotype, anthocyanin content, gene expression level, change trait

## Abstract

Sampling for DUS test of flower colors should be fixed at the stages and sites that petals are fully colored, and besides, flower colorations are uniform among individuals and stable for a period of time to allow testers to get consistent results. It remains a problem since spatial and temporal flower colorations are reported a lot but their change traits are little discussed. In this study, expression state, uniformity and stability of color phenotypes, anthocyanin contents, and gene expression levels were taken into account based on measurements at 12 development stages and three layers (inner, middle, and outer petals) of two varieties of *Ranunculus asiaticus* L. to get their best sampling. Our results showed that, outer petals of L9–L10 (stage 9–stage 10 of variety ‘Jiaoyan zhuanhong’) and C5–C6 (stage 5–stage 6 of variety ‘Jiaoyan yanghong’) were the best sampling, respectively. For DUS test, it is suggested to track flower colorations continuously to get the best sampling as well as representative colors since different cultivars had different change traits, and moreover, full expression of color phenotypes came later and lasted for a shorter duration than those of anthocyanin contents and gene expressions. Our innovation exists in following two points. Firstly, a model of change dynamic was introduced to illustrate the change traits of flower colorations, anthocyanin contents, and gene expressions. Secondly, genes used for expression analysis were screened on account of tentative anthocyanins, which were identified based on comparison between liquid chromatography–mass spectrometry (LC–MS) results and molecular mass and mass fragment pattern (M^2^) of each putative anthocyanin and their fragments deduced in our previous study. Gene screening in this regard may also be interest for other non-model plant genera with little molecular background.

## 1. Introduction

Anthocyanins are water-soluble pigments with a backbone structure of C6-C3-C6 (A-ring-C3-bridge-B-ring), giving fruits, flowers, seeds or other plant organs marvelous colors of pink, red, purple, blue, etc. [1,2]. Anthocyanin biosynthesis includes phase of backbone forming and phase of anthocyanidin modification. Anthocyanidins (cyanidin, delphinidin, and pelargonidin), backbones of anthocyanins, are inherently unstable and colorless, and can be modified with glycosyl-, methyl-, and acyl-groups (glycosylation, methylation, and acylation) [3,4]. Modification can turn anthocyanidins into stable and colorful anthocyanins.

*Ranunculus asiaticus* L. has great diversity in flower colorations and is a suitable model for studying of flower colorations [5,6]. Our previous studies have established the pathway of anthocyanin biosynthesis of *Ranunculus asiaticus* L., and revealed that anthocyanin biosynthesis of this plant was relatively complete with three branches of F3H, F3′H, and F3′5′H and three modifications of glycosylation, methylation, and acylation [5]. Complete branches and modifications can explain its great coloration diversity, including: (1) petal colors vary from common hues (white, yellow, orange, pink, red, purple, etc.) to rare hues (green, blue purple, purple black, brown, etc.); (2) gradual colorations of the same hue are common among varieties; (3) there are types of mono-, bi- and tri-colored, as well as various distribution of secondary or tertiary color [7]. Our previous studies have also concluded there are 144 putative anthocyanins in *Ranunculus asiaticus* L. [5].

Flower color is a leading characteristic of DUS test (D: Distinctness, inter-cultivar variation; U: Uniformity, intra-cultivar homogeneity; S: Stability, homogeneity of generations) [7,8]. DUS test is the basis for the granting of plant breeders’ rights (PBR) [9,10]. It is of great importance to test flower color in a proper way. However, testers are puzzled by the sampling (i.e., which development stage, which layer of petals) for DUS test of flower colors since flower coloration is a course of spatial and temporal change [11,12]. Such confusion is worse when it comes to double flowers particularly whose anther condition is unavailable to achieve an agreed stage for DUS test. Different sampling leads to obviously different results, which definitely influences the accuracy of DUS test and variety description, and then, harm the marketing and PBR protection. On the other hand, sampling cannot be casually fixed at certain stage. Sampling for DUS test should take the expression state, stability and uniformity of characteristics into account, i.e., sampling should be defined at the stages and sites that petals are fully colored while the coloration is relatively stable for a period of time and uniform among individuals to allow testers to get consistent results [7]. However, how to translate this requirement into practical protocol remains a big problem.

There are lots of studies on spatial and temporal changes of flower colorations [13,14,15]. Along with flower growth, petal colors of most plants change with lightness increasing while chromaticity decreasing, e.g., ornamental crabapple, pansy, *Cymbidium* orchid, *Paeonia lactiflora* Pallas, et al. [16,17,18,19]. However, detailed change traits of flower coloration vary greatly among different plant species, even among different varieties of the same plant species [16,17,18,19]. Therefore, regular change pattern in common is difficult to conclude. On the other hand, previous studies did not investigate uniformity of flower colorations among individual plants, and particularly, they did not reveal change traits of flower colorations, including change speed, tendency, status, so stability of flower coloration during a given interval is unknown yet. Therefore, it cannot be derived which stages and which layers of petals are best for DUS test.

In this study, based on two varieties of double flowers with representative colors, we researched expression states of flower coloration at 12 flower development stages and three layers (inner, middle, and outer petals), and meanwhile, coloration stability during a given interval as well as uniformity among individual plants were also investigated with the aid of Model of Change Dynamic and Standard Deviation values, respectively. Combined with evidences of anthocyanin content and gene expression patterns, best sampling for DUS test of flower coloration of the two varieties was concluded. For DUS test, it is suggested to track flower coloration continuously to get the best sampling as well as representative color. In our study, a model of change dynamic was introduced to illustrate the change traits of flower colorations, anthocyanin contents, and gene expressions, including their change speed, tendency, and status. Furthermore, genes used for expression analysis were screened based on tentative anthocyanins. Tentative anthocyanins were identified on account of comparison between LCMS results and molecular mass and mass fragment pattern (ms^2^) of each putative anthocyanin and their fragments which were deduced in our previous research [5]. Our study will provide unique and novel insights into the spatial and temporal changes of flower colorations not only from the view of expression state, uniformity, and stability, but also providing basis for DUS test sampling of flower colors.

## 2. Results

### 2.1. Spatial and Temporal Change Traits of Flower Coloration

#### 2.1.1. Expression States of Flower Coloration

**Figure 1:**
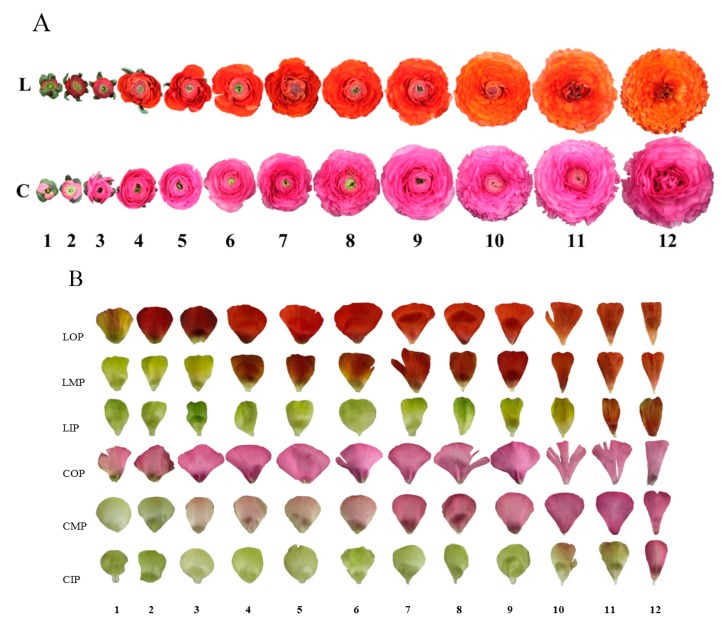
Flowers and petals at 12 development stages and three layers of two varieties of *Ranunculus asiaticus* L. (**A**) flowers; (**B**) petals; L: ‘Jiaoyan zhuanhong’; C: ‘Jiaoyan yanghong’; 1–12: stage 1–stage 12, time interval between stage 1 and stage 12 is two weeks, among which, intervals between two adjacent stages are two days from stage 1 to stage 4 and one day from stage 4 to stage 12; LOP: outer petals of ‘Jiaoyan zhuanhong’; LMP: middle petals of ‘Jiaoyan zhuanhong’; LIP: inner petals of ‘Jiaoyan zhuanhong’; COP: outer petals of ‘Jiaoyan yanghong’; CMP: middle petals of ‘Jiaoyan yanghong’; CIP: inner petals of ‘Jiaoyan yanghong’.

**Figure 2:**
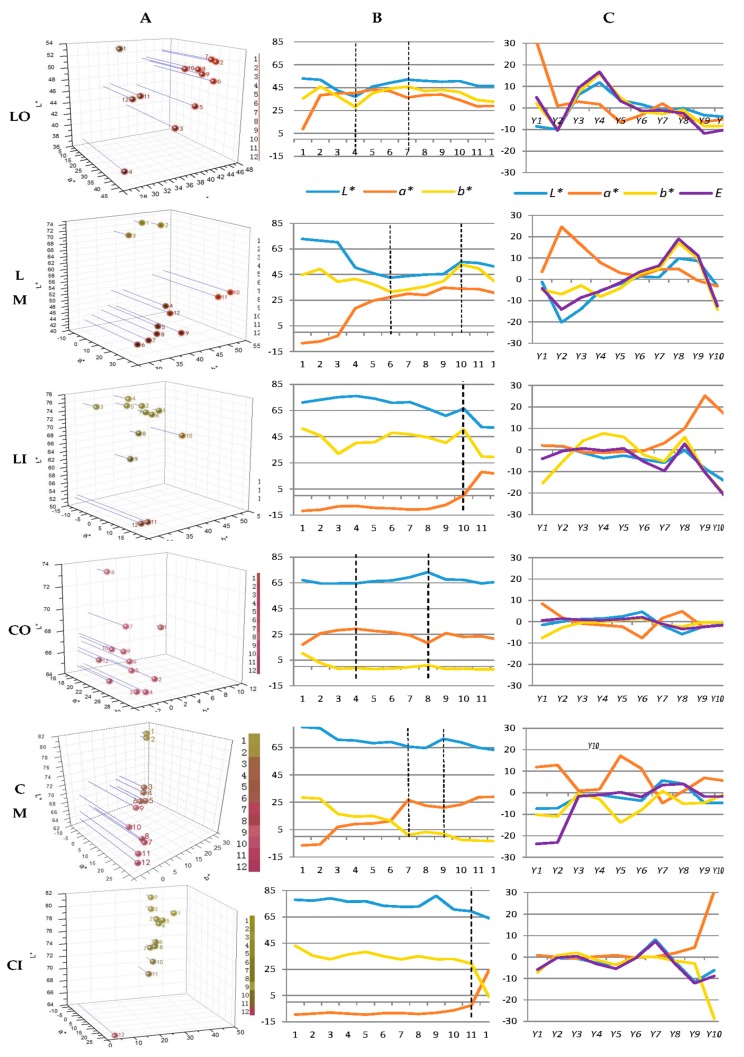
Colorations and corresponding model of change dynamics of petals of three layers at 12 development stages. L-O, L-M, and L-I: outer, middle, and inner layers of petals of ‘Jiaoyan zhuanhong’, respectively; C-O, C-M, and C-I: outer, middle, and inner layer of petals of ‘Jiaoyan yanghong’, respectively; (**A**) CIE systems of colorations, in each individual figures, balls named 1–12 reflected the colors of the 12 development stages (Figure 1), and corresponding color chars were shown in the right; (**B**) measurements of L*, a* and b* at 12 stages; (**C**) model of change dynamics of petal colorations: $Y_{jv}=\int_{j}^{j+1}\left[v_{pj}+\left(t-t_{j}\right)\cdot \left(v_{p,j+1}-v_{pj}\right)/(t_{j+1}-t_{j})\right]dt\cdot \frac{\varepsilon }{1+e^{-a_{pj}}}$, where measurements of L*, a*, b* as well as E* of two varieties were recorded as *l* (Jiaoyan zhuanhong) or *c* (Jiaoyan yanghong), i.e., $l_{p,\;j+1}=l_{p}\left(t_{j+1}\right),\;\left(p=\mathrm{outer}\;\mathrm{petal},\;\mathrm{middle}\;\mathrm{petal}\;\mathrm{or}\;\mathrm{inner}\;\mathrm{petal};j+1=\mathrm{stage}\;1,\ldots ,12\right)$; $v_{pj}=\frac{l_{p,j+1}-l_{pj}}{t_{j+1}-t_{j}};\;a_{pj}=\left|\frac{v_{p,j+1}-v_{pj}}{t_{j+1}-t_{j}}\right|$; Y1–Y10: corresponded to stage 3–stage 12; negative or positive values of $Y_{jv}$ indicated corresponding status of coloration changes were decreasing or increasing, respectively; big $Y_{jv}$ indicated the tendency was toward great changes while small $Y_{jv}$ meant changes became gentle.

Petal colorations of 12 development stages and three layers (outer, middle, and inner) were investigated (Figure 1). In terms of tones of outer petals of variety L, lightness decreased while redness accumulated rapidly during L1–L4; condition was opposite during L4–L7 where yellowness accumulated, causing the increase of lightness and decrease of redness; lightness and redness kept stable during L7–L10 and then decreased markedly along with petal aging after L10. As for variety C, accumulation of redness and blueness mainly took place in early stages (C1–C4), while redness and blueness decreased during C4–C8, and then lightness and redness decreased along with petal aging after C8. Compared with variety L, coloration changes of outer petals of variety C were less obvious. Similar change trends of outer petals of both varieties also applied to middle petals, while corresponding timings were postponed to L6 and L10, C7 and C10, respectively. When it comes to inner petals, both varieties had stable colorations with greenness and yellowness in most stages, and redness accumulated suddenly after L10 and C11, respectively (Figure 1 and Figure 2A,B).

Chromaticity (C*) indicates color tones and saturation, and therefore, can be used to reveal the expression state of color [18]. As for variety L, C* of outer petals was higher than those of middle and inner petals during L2–L9; outer petals faded in following stages while C* of middle petals rose dramatically. Conditions were different when it comes to variety C, inner and middle petals had high C* due to the high greenness in early stages and rapid shift to redness in late stages. Therefore, outer petals of both varieties had representative colors. Among the 12 stages of outer petals, L5–L10 and C3–C6 had relative high chromaticity, indicating petals at those stages had full expression of flower colors (Figure 2A, Table 1).

#### 2.1.2. Uniformity of Flower Coloration

In general, outer petals of both varieties had lower standard deviation of L*, a*, and b* than middle and inner petals at each stage. Therefore, colors of outer petals among individual plants were more uniform. Particularly, L7–L9 and C5–C11 of outer petals had the lowest SD values of all L*, a*, and b* values (Table 1).

#### 2.1.3. Stability of Flower Coloration

Previous studies applied $\Delta E^{*}$ ($\Delta E^{*}=\sqrt{{\left(\Delta L^{*}\right)}^{2}+{\left(\Delta a^{*}\right)}^{2}+{\left(\Delta b^{*}\right)}^{2}}$) to illustrate changes of flower coloration. However, $\Delta E^{*}$ cannot reveal the trait of coloration change, including change status, tendency, and cannot illustrate dynamic of coloration changes either. Therefore, we introduced model of change dynamic from marketing management [19,20,21] and made some alteration where absolute values of $a_{ij}$, instead of original values of $a_{ij}$, were used when calculating tendency of coloration changes since positive or negative values here in our study had no relation with better or worse performance, while big or small values can represent the accelerated changes or decelerated changes.

Based on measurements of L*, a*, and b*, as well as calculations of C* and E*, overall change dynamics were gained. $Y_{v}^{{}^{\circ}p}$ values of L*, a*, and b* were all negative, positive, and negative, respectively, indicating overall lightness of outer, middle, and inner petals of both varieties decreased, while redness and blueness increased. $Y_{v}^{{}^{\circ}p}$ values of C* of outer petals of both varieties were positive (1.7 and 7.7, respectively), indicating color saturation of outer petals increased. However, $Y_{v}^{{}^{\circ}p}$ values of C* of inner petals of both varieties were negative (−29.6 and −28.2, respectively) due to their original greenness and fading of greenness in following stages. Moreover, $Y_{v}^{{}^{\circ}p}$ values of E* of outer, middle and inner petals of both varieties were all negative since E* takes lightness and color saturation into account, and in our cases, decrease of lightness overtook increase of color saturation.

As for outer petals of variety L, redness/greenness had the biggest absolute values of $Y_{v}^{{}^{\circ}P}$ (12.5), indicating overall change of redness/greenness was much greater than lightness and yellowness/blueness, and the change was toward redness. In case of outer petals of variety C, overall change of yellowness/blueness was the biggest ($Y_{v}^{{}^{\circ}p}$: −12.8), and the change was toward blueness. Therefore, changing toward redness and blueness were the major factors deciding coloration changes of outer petals of variety L and C, respectively.

Absolute values of $Y_{v}^{{}^{\circ}p}$ of L*, a*, b*, C*, and E* of outer petals of both varieties were all much smaller than middle and inner petals, indicating coloration of outer petals were relatively stable along with flower development, and such stability of outer petals can also be explained by the small span between measurements (L*, a*, and b*) of outer petals (Table 1, Figure 2B), as well as the obviously visible coloration changes of middle and inner petals from green to red (Figure 2A). Taking the 12 development stages into consideration, Y7 and Y8 of variety L were the lowest $Y_{jv}$, while the lowest $Y_{jv}$ of variety C were Y3 and Y4, indicating of L9–L10 and C5–C6 were the most stable stages among the 12 stages, respectively.

### 2.2. Metabolic Evidence of Anthocyanin Content for the Change Traits of Flower Colorations

**Figure 3:**
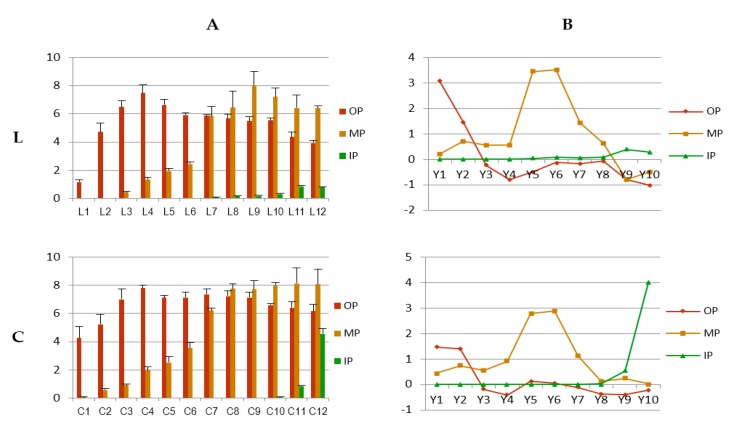
Anthocyanin content and corresponding model of change dynamics of petals of three layers at 12 development stages of both varieties. L: variety ‘Jiaoyan zhuanhong’; C: variety ‘Jiaoyan yanghong’; OP, MP and IP: outer, middle and inner layer of petals, respectively; (**A**) anthocyanin content measured using pH differential method and displayed with unit (mg 100 g^−1^ FW), L1–L12, C1–C12: measurements of petals at 12 development stages (Figure 1); (**B**) Model of Change Dynamics of anthocyanin content: $Y_{jv}=\int_{j}^{j+1}\left[v_{pj}+\left(t-t_{j}\right)\cdot \left(v_{p,j+1}-v_{pj}\right)/(t_{j+1}-t_{j})\right]dt\cdot \frac{\varepsilon }{1+e^{-a_{pj}}}$, where measurements of anthocyanin content of two varieties were recorded as *l* (Jiaoyan zhuanhong) or *c* (Jiaoyan yanghong), i.e., $l_{p,\;j+1}=l_{p}\left(t_{j+1}\right),\;\left(p=\mathrm{outer}\;\mathrm{petal},\;\mathrm{middle}\;\mathrm{petal}\;\mathrm{or}\;\mathrm{inner}\;\mathrm{petal};\;j+1=\mathrm{stage}\;1,\ldots ,12\right)$; $v_{pj}=\frac{l_{p,j+1}-l_{pj}}{t_{j+1}-t_{j}};\;a_{pj}=\left|\frac{v_{p,j+1}-v_{pj}}{t_{j+1}-t_{j}}\right|$; Y1–Y10: corresponded to stage 3–stage 12; negative or positive values of $Y_{jv}$ indicated change status of anthocyanin content were decreasing or increasing, respectively; big $Y_{jv}$ indicated the tendency was toward great changes while small $Y_{jv}$ meant changes became gentle.

To confirm the temporal and spatial change traits of color phenotypes, corresponding change traits of anthocyanin contents were investigated. Total anthocyanin (TA) of outer, middle, and inner petals at 12 development stages were measured. Figure 3A showed that, overall anthocyanin contents of outer petals of both varieties were higher than middle petals and inner petals, giving evidence for the full expression of coloration of outer petals illustrated in 3.1.1. Similarly, small values of $Y_{v}^{{}^{\circ}p}$ of outer petals of both varieties revealed anthocyanin accumulation of outer petals was more stable than those of inner and middle petals during the 12 stages.

In terms of outer petals of both varieties, negative values of both Y3 indicated anthocyanin content decreased at L5 and C5 (Figure 3B). Meanwhile, small absolute values of Y3–Y8 of variety L indicated anthocyanin content kept stable during L5–L10 (Figure 3B). Such stability was also available to variety C, which was more durable and lasted from C5 to C12 (Figure 3B). The stability may be caused by the balance between increase of anthocyanin content and petal growth. Besides, measurements at above stages (L5–L10; C5–C12) had smaller SD than other stages on the whole, revealing good uniformity among individuals during these stages (Figure 3A).

Change traits of anthocyanin contents generally matched with those of color phenotypes. Growth trends of anthocyanin contents were comparable to those of chromaticity (C*). Fluctuation of color phenotypes of middle petals of both varieties was much larger than those of outer or inner petals, which were also supported by data of anthocyanin contents. However, full expression of anthocyanins came earlier and lasted longer than those of flower colorations.

### 2.3. Molecular Evidence of Gene Expression Patterns for the Change Traits of Flower Colorations

#### 2.3.1. Selecting of Reference Genes for Quantitative Real-Time PCR

Based on results of coefficient of variation, geNorm and Normfinder, *β-actin* and *GAPDH* showed good stability (CV: 3.85 and 4.33, respectively; M value: 0.34 and 0.42, respectively; S value: 0.085 and 0.073 respectively).

Optimal number of combination of reference genes was 2 based on pairwise variation (Vn/n+1) where V2/3 was 0.142. Therefore, *β-actin* and *GAPDH* were combined as endogenous control.

#### 2.3.2. Gene Screening for Expression Pattern Analysis

We have established pathway of anthocyanin biosynthesis of *Ranunculus asiaticus* L., and 176 unigenes involved in the pathway were revealed based on transcriptome and gene expression patterns in corresponding colored varieties (Table 2) [16]. Among these unigenes, 102 unigenes were related to backbone forming of anthocyanidins (cyanidin, delphinidin and pelargonidin) and 74 unigenes were responsible for anthocyanidin modification (Table 2) [16].

**Figure 4:**
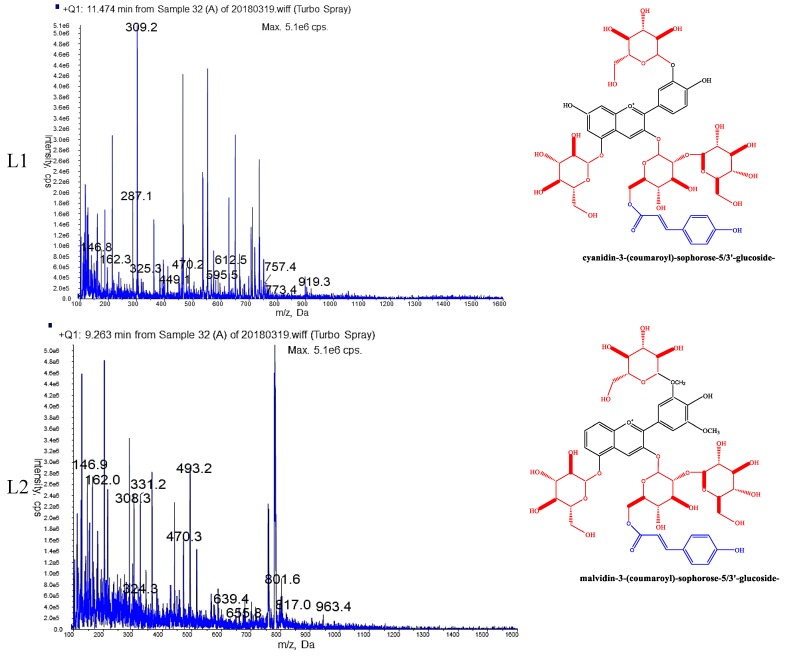

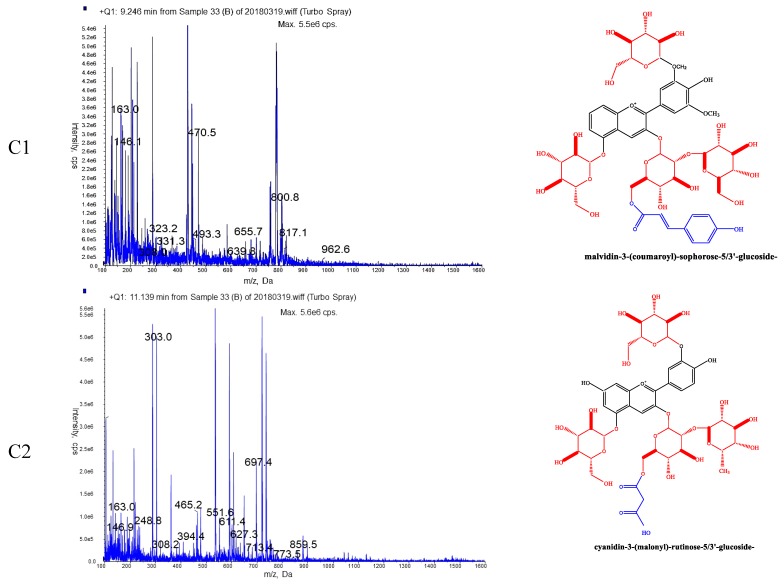
HPLC–MS chromatograms and corresponding structures of major anthocyanins of two varieties of *Ranunculus asiaticus* L. L1 and L2 were the major anthocyanins of variety ‘Jiaoyan zhuanhong’; C1 and C2 were the major anthocyanins of variety ‘Jiaoyan yanghong’.

Based on these unigenes and corresponding pathway, around 144 putative structures of anthocyanins can be inferred. Tentative identification of major anthocyanins of the two varieties were concluded based on the comparison between LCMS results and molecular mass and mass fragment pattern (ms^2^) of each putative anthocyanin (Figure 4). It is important to note that, different from conventional comparison, we compared LC–MS results with molecular mass of each putative anthocyanin and their fragments. Details are as follows, for each putative anthocyanin, we listed molecular masses of all possible fragments and set values of those molecular masses as screening conditions. After screening, component peaks with the set values can be found, indicating this putative anthocyanin can be deduced as tentative one in corresponding variety. In case of nothing left after screening, deducing cannot be made accordingly. In some cases, fragment position cannot be fixed. Taking compounds L1 and C2 as examples, a ‘glycoside’ can be deduced in L1 and C2 judging from the fragment molecular mass of ‘162′ (Figure 4). However, such glycosylation may be in their C3′, or in their C5 since 3′GT and 5GT of *Ranunculus asiaticus* L may be responsible for C3′-, C5-glycosylation (Figure 4, Table 2). In contrast, the glycosylation of compounds L2 or C1 would be in their C5, rather than C3′, since their C3′ were occupied by methyl- group (Figure 4). For a collection of 144 putative anthocyanins, it is a huge job to screen individually, but it can save expense compared with NMR.

Based on glycosyl-, methyl-, and acyl-groups of tentative anthocyanins, 45 unigenes (annotated as 3GGT, 3GT, 3AT and FMT) and 54 unigenes (annotated as 3GGT, 3GT, 3AT, FMT, 3GRT, and 3MAT) were preliminarily chosen for variety ‘Jiaoyan zhuanhong’ and variety ‘Jiaoyan yanghong’, respectively. Further screening to determine which genes might be involved in anthocyanidin modification was descripted as three steps. Firstly, unigenes with expression level below 1 during stage 2–stage 3 of outer petals were excluded. Secondly, expression levels of unigenes in outer petals were much higher (more than four-fold) than inner petals during stage 1–stage 5 were kept. Thirdly, expression levels of unigenes in outer petals were higher (more than two-fold) than middle petals during stage 1–stage 3 were kept. The screening resulted in one unigene encoding 3GT for variety L, and two unigenes encoding 3GT and 3MAT for variety C. The reason why two different *3GTs* were screened may be that major anthocyanin structures of the two varieties were different, e.g., C3 glycosylation, C6′′ acylation, et al., and the two varieties may apply different *3GT* genes for C3 glycosylation. 

#### 2.3.3. Change Traits of Expression Levels of *c72570*, *c130622*, and *c83020*

**Figure 5:**
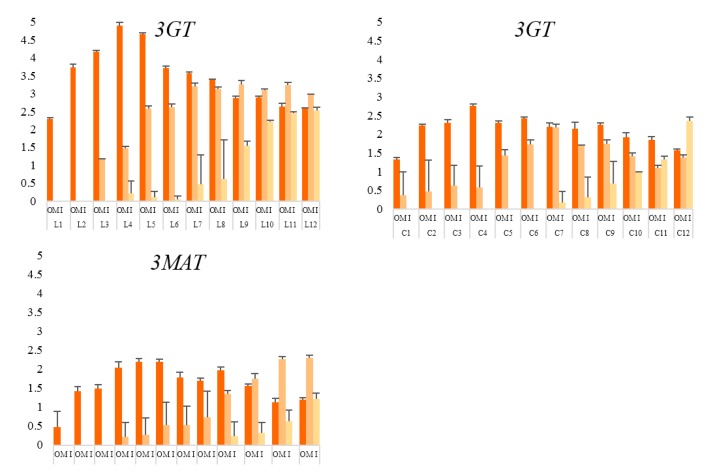
Gene expression patterns of two varieties of *Ranunculus asiaticus* L. L1–L2: stage 1–stage 12 of ‘Jiaoyan zhuanhong’ (Figure 1); C1–C12: stage 1–stage 12 of ‘Jiaoyan yanghong’; O: outer petal; M: middle petal; I: inner petal; *c72570*, *c130622*, and *c83020* were unigenes annotated as encoding 3GT, 3GT, and 3MAT based on transcriptome sequencing.

**Figure 6:**
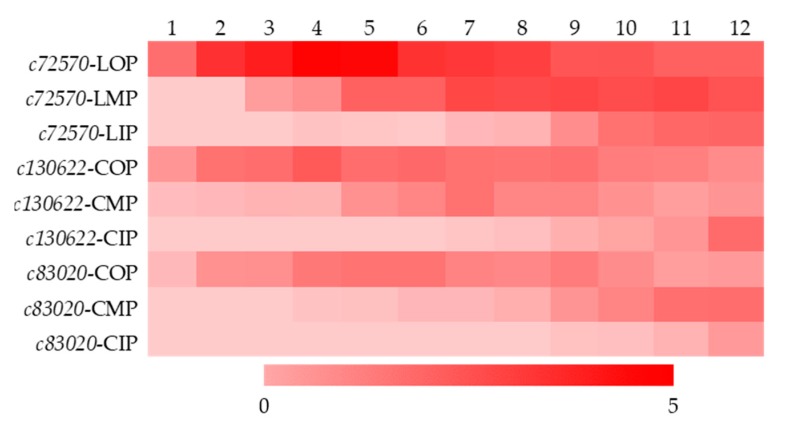
Heat map of gene expression patterns. 1–12: stage 1–12 (Figure 1); *c72570*, *c130622*, and *c83020* were unigenes annotated as encoding 3GT, 3GT, and 3MAT based on transcriptome sequencing; LOP, LMP, and LIP stood for outer petals, middle petals, and inner petals of variety ‘Jiaoyan zhuanhong’; COP, CMP, and CIP stood for outer petals, middle petals, and inner petals of variety ‘Jiaoyan yanghong’. The darker the color of the square within the heat map, the higher the expression level.

Based on selecting of reference genes, gene expression patterns were analyzed (Figure 5). Heat maps showed that gene expression levels of outer petals of both varieties were much higher than middle and inner petals, giving another evidence for the fully expressed color in outer petals. Expression peaks of the three genes in outer petal were during L3–L5 and C3–C6, earlier than expression of color phenotype (Figure 6).

Expression levels of three genes of outer petals had smaller SD values than those of middle and inner petals. In outer petals, expression levels of *c72570* had small SD values during L5–L10, while expression level of *c130622* and *c83020* had small SD values during C5–C6 that, indicating L5–L10, C5–C6 were the more uniform stages in terms of gene expression levels (data not shown).

Both varieties had smaller absolute values of $Y_{v}^{{}^{\circ}p}$ in outer petals than those in middle or inner petals. Among the 12 stages of outer petals, absolute values of $Y_{jv}$ of L9–L10 and C5–C9 were smaller, indicating above stages had stable gene expression levels (data not shown).

In general, change traits of gene expression patterns matched with those of color phenotypes, as well as anthocyanin contents. Full expression of genes came earlier than those of anthocyanins and color phenotypes, and lasted for a longer duration than those of color phenotypes, but shorter than those of anthocyanins. Reasons for above difference exist in that, final coloration is decided by many factors, e.g., area size, texture of petal, categories of anthocyanins, etc. On the other hand, total anthocyanins here in our study contained intermediate anthocyanins produced in the course of degradation or synthesis.

### 2.4. Correlation among Color Phenotypes, Anthocyanin Contents, and Gene Expression Patterns

Correlations between L*, a*, b*, total anthocyanin content (TA), expression level of gene (*c72570*, *c130622*, and *c83020*) were analyzed. As for variety L, the most significant correlation was between *c72570* and a*, followed by correlations between TA and a* (Table 3), indicating *3GT* was the leading factor causing redness, and redness was the main color of anthocyanins of variety L. Gene *c72570*, *c130622*, and *c83020* had similar trends of correlations with L*, a*, and TA. Different from variety L, b* of variety C had significant correlation with L*, TA, and gene expression level, and had significant negative correlation with a* (Table 4), indicating yellowness/blueness was also the leading factor influencing color of variety C, and redness and blueness were the main color of variety C. Correlations between genes (*c72570*, *c130622*, and *c83020*) and color phenotypes can be explained by previous studies, that glycosylation and acylation add red and purple hues to flower coloration [22,23].

**Figure 7:**
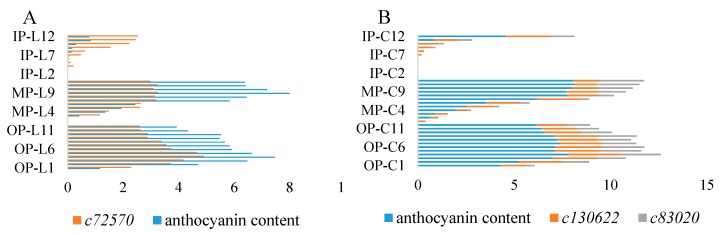
Correlation between anthocyanin contents and gene expression levels. A: measurements of ‘Jiaoyan zhuanhong’; B: measurements of ‘Jiaoyan yanghong’; IP: inner petals; MP: middle petals; OP: outer petals; L1–L12: stage 1–12 of ‘Jiaoyan zhuanhong’; C1–C12: stage 1–12 of ‘Jiaoyan yanghong’; c72570, c130622, and c83020 were genes annotated as encoding 3GT, 3GT, and 3MAT.

Particularly, correlations between anthocyanin contents and gene expression levels were analyzed. It was obvious that anthocyanin contents had significant positive correlation with gene expression levels (Figure 7). Correlation between anthocyanin content of ‘Jiaoyan zhuanhong’ with *c72570* was 0.871, while correlations between anthocyanin content of ‘Jiaoyan yanghong’ with *c130622* and *c83020* were 0.845 and 0.906, respectively.

Based on above results, L9–L10 and C5–C6 of outer petals were the best sampling for DUS test of flower coloration of the two varieties.

## 3. Discussion

In the practice of DUS test, sampling for DUS test depends on testers’ experience especially when anther is unavailable to indicate the stage of fully open flowering. According to requirement of DUS test, sampling should be fixed when and where a phenotype trait is fully expressed, uniform among individuals and stable for a given duration. How to translate above requirements into practical protocol became our purpose. Since DUS test focus on phenotype traits, our study investigated the change traits of flower colorations temporally and spatially. Our results showed that, change traits of anthocyanin contents and gene expression patterns were comparable to those of flower colorations, illustrating the change traits of flower colorations on metabolic and molecular basis. Moreover, full expression of flower colorations came later and lasted for a shorter duration than those of anthocyanins and genes, further giving us more confidence to use the parameter of color phenotype in this regard.

‘Jiaoyan zhuanhong’ and ‘Jiaoyan yanghong’ share similar parent lines. They are both double flower, making it possible to investigate flower colorations spatially. Particularly, their colors are lateritious and carmine, indicating they may adopt different branches of the pathway of anthocyanin biosynthesis. In our study, the two varieties have different gene expression patterns, causing different anthocyanin accumulation, leading to different change traits of flower coloration. Such variation may also apply to different plant species. Therefore, regular change pattern in common is difficult to conclude, and it is required to track color phenotypes continuously to get the best sampling as well as the representative colors, instead of fixing the sampling. Model of change dynamic was applied for the purpose of continuous tracking. This model can illustrate the change status and tendency, and thus, reveal the stability of flower coloration for a given duration.

Our previous studies have revealed the pathway of anthocyanin biosynthesis of *Ranunculus asiaticus* L., identified enzyme-encoding genes involved in this pathway, and deduced 144 putative anthocyanin structures [16]. Based on transcript sequencing and gene annotation, binding position of glycosyl-, acyl-, or methyl-groups of the 144 structures can also be identified [16]. In this study, tentative anthocyanins of the two varieties were concluded based on the comparison between LCMS results and molecular mass of each putative anthocyanin and their fragments. Based on tentative anthocyanins, genes involved in modification phase were screened for analysis of expression patterns. Our trial design may also be interest for other non-model plant genera, especially whose molecular background are not very clear.

Change traits of expression levels of two unigenes encoding 3GT generally matched those of color phenotypes and total anthocyanin content. Since glycosylation is the first step of modification, and C3 glycosylation was very common in anthocyanin colored plants [24,25,26]. Therefore, *3GT* may be a good choice for research in this regard. Gene expression patterns discussed in previous studies focus on commonly used genes at crosspoint of upstream of anthocyanin biosynthesis [27,28,29], e.g., *CHS*, *DFR*, etc. Compared with genes involved in downstream (anthocyanidin modification), gene involved in upstream (backbone forming) have more other functions besides their roles in anthocyanin biosynthesis [30,31,32]. Besides, abundant and durable flower colorations are largely decided by the phase of modification [33,34,35,36]. That is why we focus on genes involved in anthocyanidin modification. Of course, comprehensive analysis of expression pattern of all genes involved in the pathway is more preferable.

## 4. Materials and Methods

### 4.1. Spatial and Temporal Change Traits of Flower Coloration

Two varieties of *Ranunculus asiaticus* L. (bred by ‘Brighten’, Kunming, China) were selected, namely ‘Jiaoyan zhuanhong’ (lateritious, hereinafter referred to as variety L) and ‘Jiaoyan yanghong’ (carmine, hereinafter referred to as variety C) (Figure 1).

To get uniform plants, tissue culture method [37] was applied with some modification. Bulb sprouts germinated under 10-day fully shaded and 8–10 °C condition were used as explants. After sterilization in 75% ethanol for 20 s and 0.15% HgCl_2_ for 6 min, explants were transferred to medium of ‘BA 6 mg L^−1^ + NAA 0.2 mg L^−1^’, or ‘TDZ 4.0 mg L^−1^ + NAA 0.2 mg L^−1^’ to induce callus. After two months, explants were transferred to medium of ‘1/2 MS + NAA 0.1 mg L^−1^’. Seedlings with height of 5 cm and three leaves were transferred to greenhouse condition for following growth.

Petals of the two varieties at 12 development stages and three layers (inner, middle, and outer) were sampled (Figure 1). Middle layers refer to the 11th and 16th layers of variety L and variety C counting from outside, respectively.

### 4.2. Measurement of Color

Color of samples were measured using a colorimeter (CM-700d, Japan) and reported in the CIE system (L*: lightness, a*: redness/greenness, b*: yellowness/blueness) [38]. Chromaticity ($C^{*}=\sqrt{{\left(a^{*}\right)}^{2}+{\left(b^{*}\right)}^{2}}$) and total coloration ($E^{*}=\sqrt{{\left(L^{*}\right)}^{2}+{\left(a^{*}\right)}^{2}+{\left(b^{*}\right)}^{2}}$) were calculated. Color was measured in the center of upper third of upper side of petals.

### 4.3. Measurement of Anthocyanin Content and Tentative Identification of Anthocyanin Components

Anthocyanin contents were measured according to the pH differential method [11,39] with some modifications [40]. 10 g petal tissues were extracted with 80% (*v/v*) ethanol (pH 1.0), and put under sonication for 30 min. This process was repeated a third time. The combined extracts were diluted with 80% ethanol (pH 1.0) to 50 mL, and centrifuged for 5 min at 10,000 rpm. 5 mL aliquot of supernatant was diluted to 50 mL with a solution of pH 1.0 KCl (0.02 mol L^−1^ KCl: 0.2 mol L^−1^ HCl (6:5, by vol)), and another 5 mL aliquot was diluted to 50 mL with pH 4.5 NaAc (water was added to 19.284 g NaAc and 16 mL acetic acid to final volume of 500 mL). Solutions were balanced for 80 min. The absorbance of both solutions was measured at 525 nm and 700 nm. Total anthocyanin content in the original sample was calculated using the formula

*A* = (*A*_525_ − *A*_700_) pH_1.0_ − (*A*_525_ − *A*_700_) pH_4.5_ (1)

Total anthocyanin content

(mg/g) = (*A* × MW × DF × *V* × 1000)/(*mɛ*) (2)

where MW is the molecular weight; DF is the dilution factor; *V* is the liquid volume, *m* is the sample mass; *ɛ* is the molar extinction coefficient.

HPLC–MS was applied to verify the anthocyanin components. 20 g petal tissue from each sample was ground with liquid nitrogen. 10 g petal powder was extracted with 10 mL methanol:water:methanoic acid (70:20:10, by vol.), and put under high-speed vortex oscillation for 10 min and sonication for 5 min. The extract was centrifuged for 5 min at 6000 rpm. 1 mL supernatant was filtrated (0.22 μm) and analyzed by LC–MS. The LC–MS system consisted of Agilent 1290 UHPLC (Aglient, Santa Clara, CA, USA), API4000 mass spectrometer (AB Sciex, Redwood, CA, USA) with a CAPCELL CORE PC (2.7 μm, 100 × 2.1 mm, Shiseido, Japan). A 5 μL aliquot of each prepared extract was separated with a mobile phase consisting of 0.1% formic acid water containing 1 mmol L^−1^ ammonium acetate (A) and acetonitrile (B) by HILIC chromatography maintained at 35 °C with a flow rate of 0.2 mL min^−1^. A gradient was applied: min/A%/B% as 0/97/3, 5/97/3, 25/10/90, 30/10/90, 32/97/3, 40/97/3. The eluent was scanned with electrospray ionization (ESI) in the positive mode from *m/z* 100–1600 amu. Data were acquired for processed with the aid of Analyst^®^ 1.6.3 Software (AB Sciex Pte. Ltd., Singpore).

Our previous research has deduced 144 putative anthocyanin components. Based on comparison of HPLC–MS results and molecular mass & mass fragment pattern (M^2^) of each putative anthocyanin, tentative anthocyanin components can be identified.

### 4.4. Expression Patterns of Related Gene

1μg RNA of plant materials was taken to reverse-transcribe the first strand cDNA (PrimeScript™ RT Reagent Kit, Takara, Dalian, China). qRT-PCR was conducted using 20 μL reaction mixtures, including 2.0 μL of cDNA, 10 nM primers and 10 μL of iTaq™ Universal SYBR Green supermix (Bio-RAD, Hercules, CA, USA), and Bio-Rad MiniOpticon Real-Time PCR (Bio-RAD, Hercules, CA, USA). Cq value and relative expression levels of genes was determined using CFX Manager 2.1 and methods described as Pfaffl et al. [41].

Suitable housekeeping genes of qPCR were screened from five candidate genes prior to gene expression analysis. Expression stabilities of candidate genes of outer, middle and inner petals of ‘Doppio Pandora-purple’ and ‘Doppio Pandora-purple’ at fully open flowering were evaluated based on coefficient of variation of Cq values, M values of geNorm (http://medgen.ugent.be/-jvdesomp/geNorm/) and S values of NormFinder (http://www.mdl.dk/publicationsNormFinder.htm). Optimal number of housekeeping genes required was assessed (based on Vn/n+1, threshold is 0.15, geNorm) [42,43,44,45].

Expression patterns were conducted on genes linked to the biosynthesis of tentative anthocyanin components. Endogenous controls were geometrical means of expression levels of housekeeping genes [46,47].

### 4.5. Change Traits and Model of Change Dynamic of Flower Coloration

Measurements of two varieties were recorded as l or c, i.e., $l_{p,\;j+1}=l_{p}\left(t_{j+1}\right),\;\left(p=\mathrm{outer}\;\mathrm{petal},\;\mathrm{middle}\;\mathrm{petal}\;\mathrm{or}\;\mathrm{inner}\;\mathrm{petal};j+1=\mathrm{stage}\;1,\ldots ,12\right).$ Measurements were used to calculate change speed: $v_{pj}=\frac{l_{p,j+1}-l_{pj}}{t_{j+1}-t_{j}},$ (1); Model of Change Dynamic: $Y_{jv}=s_{v}^{p}\left(t_{j},\;t_{j+1}\right)\cdot \lambda \left(a_{pj}\right),\;$ (2); overall change dynamic: $Y_{v}^{{}^{\circ}p}=\overset{j-1}{\underset{j=1}{\sum }}Y_{v}^{{}^{\circ}p}\left(t_{j},\;t_{j+1}\right),$ (3). Change traits can be revealed in the Model of Change Dynamic, i.e., change status: $s_{v}^{p}\left(t_{j},\;t_{j+1}\right)=\int_{j}^{j+1}\left[v_{pj}+\left(t-t_{j}\right)\cdot \left(v_{p,j+1}-v_{pj}\right)/(t_{j+1}-t_{j})\right]dt,$ (4); change tendency: $\lambda \left(a_{pj}\right)=\frac{\varepsilon }{1+e^{-a_{pj}}},\;a_{pj}=\left|\frac{v_{p,j+1}-v_{pj}}{t_{j+1}-t_{j}}\right|,$ (5). Negative or positive values of $s_{v}^{p}$ indicated corresponding status of coloration changes were decrease or increase, respectively. Since $\lambda \left(a_{pj}\right)\in 2$ if $a_{pj}\in \pm \infty$ and $\lambda \left(a_{pj}\right)\in 1$ if $\;a_{pj}\in 0$, big $\lambda \left(a_{pj}\right)$ indicated the tendency was toward great changes while small $\lambda \left(a_{pj}\right)$ meant changes became gentle [19,20,21].

Besides, measurement of color (L*, a*, b*, C*, E*), total anthocyanin content and gene expression levels were used for correlation analysis.

## Figures and Tables

**Table 1:** Color measurements of two varieties of *Ranunculus asiaticus* L.

OP	L*	a*	b*	C*	MP	L*	a*	b*	C*	IP	L*	a*	b*	C*
L1	52.9 ± 1.0	8.9 ± 0.8	35.4 ± 1.6	36.5	L1	72.8 ± 1.9	−8.5 ± 0.7	44.8 ± 1.6	45.6	L1	71.2 ± 2.5	−11.7 ± 2.0	51.1 ± 2.1	52.5
L2	51.8 ± 0.8	38.4 ± 1.3	45.9 ± 1.2	59.8	L2	71.5 ± 2.4	−7.2 ± 1.0	49.4 ± 1.5	49.9	L2	73.2 ± 2.1	−10.9 ± 1.2	45.7 ± 1.8	47.0
L3	42.3 ± 0.9	39.9 ± 1.2	37.5 ± 1.3	54.8	L3	70.2 ± 2.0	−2.9 ± 0.7	39.4 ± 1.6	39.5	L3	75.3 ± 2.2	−8.2 ± 1.2	32.0 ± 1.7	33.0
L4	37.1 ± 1.5	40.1 ± 1.3	28.1 ± 0.7	49.0	L4	50.5 ± 1.7	18.7 ± 1.4	41.6 ± 1.8	45.6	L4	76.1 ± 1.9	−8.0 ± 1.1	40.2 ± 1.8	41.0
L5	45.8 ± 2.1	43.3 ± 0.9	40.0 ± 1.0	59.0	L5	46.1 ± 1.3	24.5 ± 1.3	37.6 ± 1.8	44.9	L5	74.1 ± 2.0	−9.5 ± 1.4	40.7 ± 1.9	41.8
L6	49.2 ± 1.2	41.9 ± 1.3	44.1 ± 1.2	60.8	L6	42.6 ± 1.5	27.4 ± 1.2	31.5 ± 1.3	41.8	L6	70.8 ± 2.1	−9.8 ± 1.1	48.0 ± 1.8	49.0
L7	52.0 ± 1.1	36.3 ± 0.8	45.8 ± 0.8	58.4	L7	43.8 ± 1.7	30.0 ± 1.8	33.3 ± 1.8	44.9	L7	71.4 ± 2.0	−10.8 ± 1.0	46.8 ± 1.8	48.0
L8	50.9 ± 0.7	38.5 ± 0.6	42.2 ± 0.7	57.2	L8	45.0 ± 1.7	29.0 ± 1.2	35.8 ± 2.0	46.0	L8	66.4 ± 2.0	−10.4 ± 1.0	44.5 ± 1.6	45.7
L9	50.1 ± 0.9	38.9 ± 0.4	42.9 ± 0.7	57.9	L9	45.4 ± 1.6	34.7 ± 1.1	39.8 ± 1.2	52.8	L9	61.0 ± 2.4	−7.1 ± 1.2	40.2 ± 2.0	40.8
L10	50.7 ± 1.1	34.2 ± 0.9	41.2 ± 1.5	53.5	L10	54.9 ± 2.0	33.9 ± 1.7	52.8 ± 1.0	62.8	L10	66.4 ± 1.9	0.2 ± 0.5	50.5 ± 1.6	50.5
L11	46.6 ± 0.8	28.7 ± 1.0	34.2 ± 1.3	44.7	L11	54.0 ± 1.4	33.6 ± 1.5	49.7 ± 1.4	60.0	L11	52.3 ± 1.8	18.1 ± 1.4	30.0 ± 1.5	35.0
L12	46.3 ± 1.5	28.9 ± 0.9	32.7 ± 1.2	43.6	L12	50.9 ± 1.4	30.3 ± 1.2	38.5 ± 1.4	49.0	L12	51.7 ± 1.3	16.4 ± 1.2	29.3 ± 1.3	33.6
C1	67.0 ± 1.1	17.1 ± 1.1	10.3 ± 0.7	19.9	C1	80.0 ± 2.1	−6.5 ± 1.3	28.4 ± 1.6	29.1	C1	78.0 ± 2.0	−9.6 ± 1.1	43.0 ± 1.7	44.1
C2	64.6 ± 0.9	25.8 ± 1.3	2.7 ± 1.1	26.0	C2	79.2 ± 1.6	−5.8 ± 1.1	27.8 ± 2.0	28.4	C2	77.3 ± 2.2	−8.8 ± 1.3	35.5 ± 1.7	36.6
C3	64.5 ± 1.7	28.3 ± 1.5	−1.7 ± 0.8	28.3	C3	70.7 ± 2.0	6.9 ± 1.1	16.6 ± 1.2	17.9	C3	79.1 ± 2.0	−8.0 ± 1.0	32.7 ± 1.6	33.7
C4	64.6 ± 1.2	29.3 ± 2.0	−1.2 ±0.2	29.3	C4	70.2 ± 1.9	9.1 ± 1.0	14.5 ± 1.2	17.1	C4	76.5 ± 1.9	−8.8 ± 1.2	36.4 ± 1.7	37.5
C5	66.3 ± 0.8	27.6 ± 1.0	−2.0 ± 0.3	27.7	C5	68.2 ± 1.7	9.5 ± 1.4	15.1 ± 1.2	17.8	C5	76.9 ± 1.7	−9.6 ± 1.2	38.3 ± 1.4	39.5
C6	66.8 ± 1.0	26.5 ± 0.6	−1.5 ± 0.3	26.5	C6	69.1 ± 2.2	11.3 ± 0.6	11.4 ± 0.8	16.0	C6	73.6 ± 2.1	−8.5 ± 1.1	35.1 ± 1.3	36.1
C7	69.3 ± 0.8	24.3 ± 0.6	−0.4 ± 0.3	24.3	C7	65.7 ± 2.3	26.7 ± 1.0	1.0 ± 0.7	26.7	C7	72.8 ± 1.9	−8.3 ± 1.0	32.6 ± 1.4	33.7
C8	73.2 ± 0.7	18.3 ± 0.7	1.1 ± 0.2	18.3	C8	64.7 ± 1.6	22.5 ± 1.6	3.3 ± 0.9	22.8	C8	72.9 ± 2.0	−9.1 ± 1.0	35.0 ± 1.7	36.2
C9	67.6 ± 0.8	25.8 ± 0.6	−1.8 ± 0.2	25.9	C9	71.4 ± 2.3	21.1 ± 1.0	1.9 ± 0.8	21.2	C9	80.9 ± 1.8	−8.2 ± 1.1	32.7 ± 1.7	33.7
C10	67.2 ± 1.1	23.1 ± 0.5	−1.6 ± 0.2	23.2	C10	68.8 ± 1.8	23.3 ± 1.2	−2.4 ± 0.9	23.4	C10	70.7 ± 1.9	−6.3 ± 1.2	32.9 ± 1.8	33.4
C11	64.5 ± 1.5	23.4 ± 0.9	−2.2 ± 0.5	23.5	C11	64.9 ± 2.1	28.7 ± 1.1	−3.0 ± 1.2	28.9	C11	69.3 ± 1.4	−2.5 ± 1.2	29.4 ± 1.4	29.5
C12	66.0 ± 1.7	20.9 ± 1.5	−2.0 ± 0.4	21.0	C12	63.1 ± 1.4	29.0 ± 0.9	−3.5 ± 1.1	29.2	C12	64.1 ± 1.3	24.4 ± 1.1	4.1 ± 1.1	24.7

Note: Colors were measured in the center of upper third of upper side of petals using colorimeter (CM-700d, Japan) and reported in the CIE system (L*: lightness, a*: redness/greenness, b*: yellowness/blueness, $C^{*}:\;\sqrt{{\left(a^{*}\right)}^{2}+{\left(b^{*}\right)}^{2}}$); L1–L12: stage 1 to stage 12 of variety ‘Jiaoyan zhuanhong’ (Figure 1); C1–C12: stage 1 to stage 12 of variety ‘Jiaoyan yanghong’ (Figure 1); OP: outer petals; MP: middle petals; IP: inner petals.

**Table 2:** Enzyme-encoding genes involved in anthocyanin biosynthesis of *Ranunculus asiaticus* L. [16].

	Protein Name	Gene Name	Unigene Number ^1^
Backbone forming	chalcone synthase	*CHS*	13 (9)
	chalcone isomerase	*CHI*	3 (3)
	flavanone 3-hydroxylase	*F3H*	32 (21)
	flavanoid 3′-hydroxylase	*F3′H*	5 (1)
	flavanoid 3′5′-hydroxylase	*F3′5′H*	9 (6)
	dihydroflavonol 4-reductase	*DFR*	39 (27)
	anthocyanidin synthase	*ANS*	1 (1)
Anthocyanidin modification	anthocyanidin 3-*O*-glucoside-2′′-*O*-glucosyltransferase	*3GGT (UGAT)*	20 (2)
	anthocyanidin 3-*O*-glucosyltransferase	*3GT (GT, UFGT)*	18 (1)
	anthocyanidin 5,3-*O*-glucosyltransferase	*3,5GT (GT1)*	6 (0)
	anthocyanidin 3-*O*-glucoside-2′’-*O*-xylosyltransferase	*3G2′′GT (UGT79B1, A3G2XYLT)*	6 (3)
	anthocyanidin-3-*O*-glucoside-2′′-*O*-rhamnosyltransferase	*3GRT (RT)*	6 (2)
	anthocyanidin-5-*O*-glucosyltransferase	*5GT (HGT8, UGT75C1)*	4 (0)
	anthocyanidin-3-*O*-glucoside-3′-*O*-beta-glucosyltransferase	*3′GT*	1 (0)
	anthocyanin 3-*O*-glucoside-6′′-*O*-coumaroyltransferase	*3AT*	5 (0)
	anthocyanin 3-*O*-glucoside-6′′-*O*-malonyltransferase	*3MAT*	3 (0)
	anthocyanin 5-*O*-glucoside-6′′′′-aromatic acyltransferase	*5AT*	3 (0)
	flavonoid *O*-methyltransferase	*FMT*	2 (0)

Note: unigene number ^1^: figures outside brackets are the number of unigenes annotated in at least 1 database, while figures inside brackets are the number of unigenes annotated in two databases.

**Table 3:** Correlations between measurements of color, total anthocyanin content, and gene expression of variety L of *Ranunculus asiaticus* L.

	L*	a*	b*	TA
a*	−0.905 **			
b*	0.487 **	−0.257		
TA	−0.783 **	0.914 **	−0.110	
*c72570*	−0.905 **	0.944 **	−0.302	0.871 **

Note: **: correlation is significant at the 0.01 level; L*: lightness; a*: redness/greenness; b*: yellowness/blueness; TA: total anthocyanin content; *c72570* was unigenes annotated as encoding 3GT based on transcriptome sequencing.

**Table 4:** Correlations between measurements of color, total anthocyanin content, and gene expression of variety C of *Ranunculus asiaticus* L.

	L*	a*	b*	TA	*c130622*
a*	−0.867 **				
b*	0.825 **	−0.984 **			
TA	−0.777 **	0.960 **	−0.960 **		
*c130622*	−0.782 **	0.893 **	−0.876 **	0.845 **	
*c83020*	−0.697 **	0.871 **	−0.862 **	0.906 **	0.797 **

Note: **: correlation is significant at the 0.01 level; L*: lightness; a*: redness/greenness; b*: yellowness/blueness; TA: total anthocyanin content; *c130622* and *c83020* were unigenes annotated as encoding 3GT and 3MAT based on transcriptome sequencing.
